# Potential anti-inflammatory effect of L-carvone in endothelial dysfunction triggered by uremia related to chronic kidney disease

**DOI:** 10.1007/s11033-026-12806-7

**Published:** 2026-09-28

**Authors:** Guilherme Miniskiskosky, Regiane Stafim da Cunha, Paulo Cézar Gregório, Carolina Amaral Bueno Azevedo, Vanessa Crocetti Pereira, Edneia Amancio de Souza Ramos, Andréa Emilia Marques Stinghen

**Affiliations:** 1 Experimental Nephrology Laboratory, Basic Pathology Department, Universidade Federal do Paraná, Curitiba, Paraná Brazil; 2 Laboratory of Human Pathology, Basic Pathology Department, Universidade Federal do Paraná, Curitiba, Paraná Brazil

**Keywords:** chronic kidney disease, monoterpene, L-carvone, inflammation biomarkers, endothelial disfunction

## Abstract

**Background:**

Chronic kidney disease (CKD) is one of the leading causes of endothelial dysfunction and directly associated with the development of cardiovascular diseases (CVD). The accumulation of uremic toxins stimulates development of an exacerbated inflammatory state, especially in the vascular endothelial microenvironment. L-carvone (CRV) monoterpene is a natural product with several described biological activities, including atheroprotective and anti-inflammatory effects. This study aimed to investigate the in vitro anti-inflammatory effect of CRV on human endothelial cells exposed to hemodialysis patients’ serum.

**Methods And Results:**

CRV did not affect endothelial cell viability at a concentration of 100 µM, either alone or in combination with uremic serum (US), but it induced a significant increase in cell proliferation (*P* < 0.05). CRV significantly reduced the gene (*P* < 0.05) and protein (*P* < 0.01) expression of Interleukin (IL) 1β (IL-1β) under uremic conditions. CRV also significantly decreased protein levels of other inflammatory biomarkers such as IL-6, IL-8, vascular endothelial growth factor (VEGF-A), serum amyloid A (SAA) (*P* < 0.01) and tumor necrosis factor α (TNF-α) (*P* < 0.05) in uremic conditions. Monocyte chemoattractant protein 1 (MCP-1) production was additionally reduced (*P* < 0.01) and consequently monocyte migration was reduced (*P* < 0.05) with CRV under uremic conditions. Cell morphology appeared to remain unchanged in the presence of CRV, and it reduced the cellular damage in the uremic environment caused by uremia.

**Conclusions:**

Altogether, these findings suggest the beneficial anti-inflammatory effect of CRV in the vascular environment in response to uremic damage in CKD patients, opening perspectives for the study of new targets to better understand the endothelial protection mechanisms.

**Graphical Abstract:**

**Figure:**
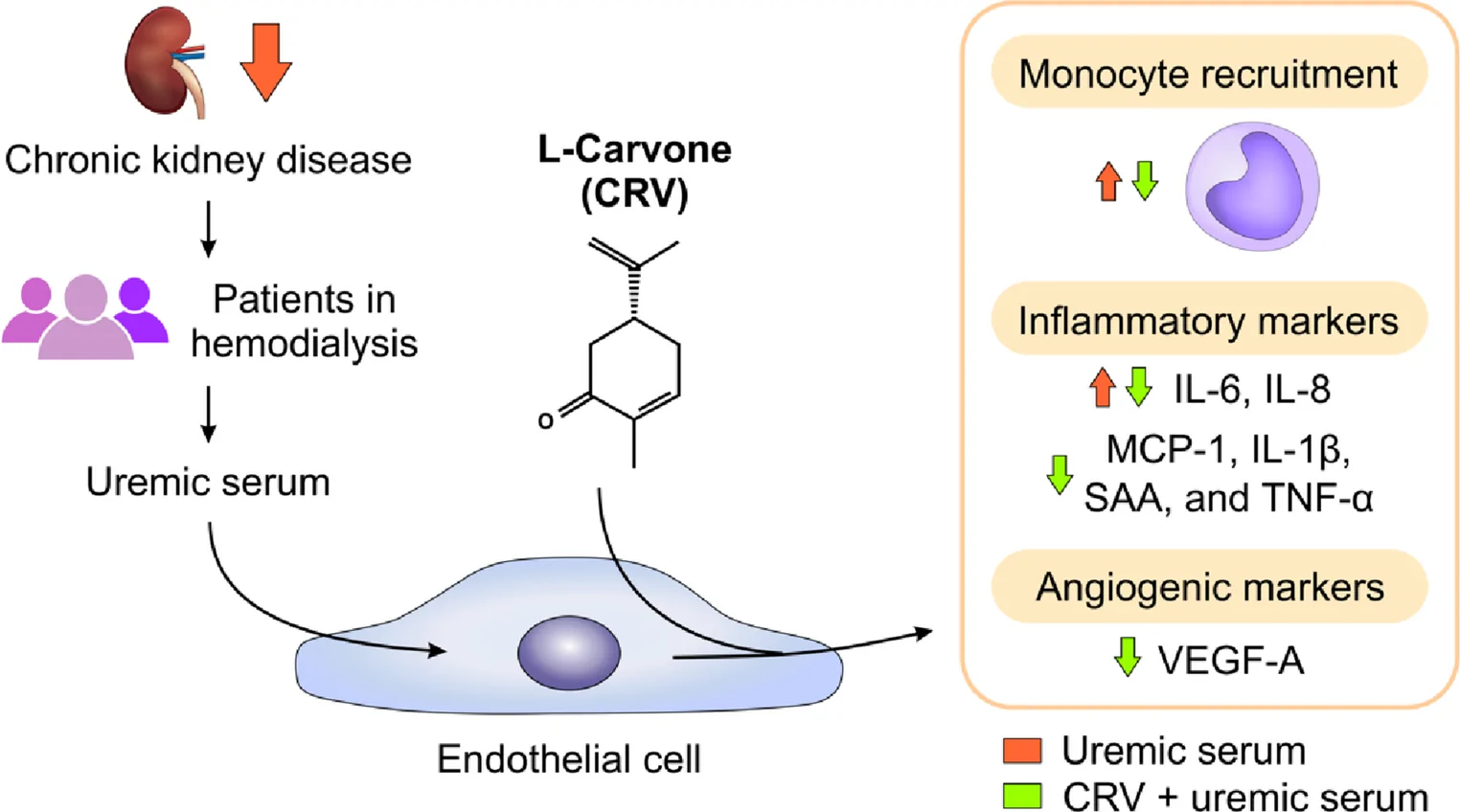

## Introduction

Chronic kidney disease (CKD) is a persistent and growing challenge for healthcare systems. According to data from Global Burden of Disease (GBD), it is the 12th leading cause of death among 133 diseases, with an incidence of 9.1% globally [1, 2]. This high CKD incidence is associated with the rising prevalence of diabetes mellitus, obesity, and hypertension, as well as the increase in the aging population [3]. CKD is also an important risk factor for the development of cardiovascular diseases (CVD) [1].

As CKD progresses, there is an accumulation of uremic toxins, particularly protein-bound toxins which are not efficiently removed in dialytic therapies [4]. The uremic state in CKD patients is often described as one of the main causes of vascular endothelial dysfunction [5]. The interaction of uremic serum and endothelial cells induces dysfunctional endothelial action leading to an inflammatory state, increasing several pro-inflammatory serum biomarkers such as interleukins 6 (IL-6) and 8 (IL-8) cytokines, adhesion molecules like vascular cell adhesion molecule 1 (VCAM-1), intercellular adhesion molecule 1 (ICAM-1), monocyte chemoattractant protein 1 (MCP-1), and E-selectin, in addition to other molecules related to endothelial activation and inflammatory cells recruitment [6, 7]. Notably, uremic toxins also induce changes in cell morphology and disrupt cell-to-cell junctions, especially adherents and occludent junctions which ultimately leads to enhanced endothelial permeability and vascular damage [8].

Several natural compounds have been described as potential drugs in pathological contexts [9]. Carvone is a ketone monoterpene primarily found in plants of the *Mentha* genus. It can be obtained in two optical isomers with distinct activities: (R)- (−) or L-carvone and (S)- (+) or D-carvone [10]. This compound exhibits a range of biological activities, including antibacterial [11], antifungal [12], antioxidant [13], anti-neurodegenerative [14], vasorelaxant [15] and anticancer effects [16, 17], in addition to its anti-inflammatory properties [15]. Some studies have related CRV in CVD protection and atheroprotective by different mechanisms, such as reducing foam cell production, modulation of macrophage balance and reducing oxidative stress [18–20].

The present study aims to investigate the anti-inflammatory effect of CRV on human endothelial cells exposed to a uremic environment. In turn, to understand its role as a potential therapeutic strategy linked to endothelial dysfunction, allowing us to gain new perspectives in the development of cardiovascular complications in CKD patients.

## Materials and methods

### Patients and Uremic serum pool preparation

A total of 14 patients with stable end-stage kidney disease on hemodialysis were selected from a single dialysis unit (Evangélico Hemodialysis Facility, Curitiba, Brazil). The inclusion criteria were patients who were on hemodialysis for at least 3 months and patients who were older than 18 years of age. The exclusion criteria were: patients who exhibited an active inflammatory or infectious disease; patients with cancer, autoimmune disease, or hepatic dysfunction; patients who were unstable with an expected survival of less than one year; patients who exhibited intact parathyroid hormone (iPTH) levels > 600 pg/mL, hypercalcemia (serum calcium > 10.3 mg/dL), and/or hyperphosphatemia (serum phosphate > 5.5 mg/dL); patients who were previous users or are current users of cinacalcet, nutritional or active forms of vitamin D, anti-osteoporotic medications, glucocorticoids, or immunosuppressive drugs; patients who had a previous history of parathyroidectomy; and patients who were pregnant [21]. The study was reviewed and approved by the Ethics Committee of Pontifícia Universidade Católica do Paraná (CEP/PUC-PR no. 1.682.929). All patients provided signed informed consent. Serum was collected from blood samples, aliquoted, and stored at − 80 °C for further analysis. The uremic serum (US) pool used in the experiments was prepared from equal amounts of serum from all patients included in the study. No samples were excluded during the pooling process, and no stratification or selection based on clinical or biochemical parameters was performed, in order to minimize the influence of individual-specific effects and obtain an averaged uremic milieu.

### Endothelial cell culture and treatments

The human endothelial cell line EA.hy926 (American Type Culture Collection [ATCC] CRL-2922, Manassas, VA, USA) was cultured in DMEM (Gibco, Waltham, MA, USA) supplemented with 10% FBS (Gibco, Waltham, MA, USA) and 10 mg/mL penicillin/streptomycin (Gibco, Waltham, MA, USA). The cells were maintained at 37 °C in a humidified atmosphere containing 5% CO_2_. For the treatments, the cells were exposed to a US pool (US) at 10% (v/v); L-carvone (CRV) (Sigma Aldrich, St Louis, MO, USA) at 100 µM; US pool and L-Carvone (US + CRV) (10% US and 100µM CRV) for 24 h. The 24 h exposure period was selected to allow sufficient time for transcriptional and inflammatory responses to uremic stimuli and CRV treatment. We performed a viability assay to determine the appropriate CRV dose for the subsequent experiments using a dose-response curve with concentrations of 100, 200, 300 400, and 500 µM, including a vehicle control group (0.01% DMSO).

### U-937 cell culture

U-937 cells (CRL-1593.2; ATCC) were purchased from a commercial tumor cell line. These cells are accepted as proxies for circulating monocytes, which are difficult to obtain in satisfactory amounts from donors’ total blood. The U-937 cells were cultured in RPMI 1640 (Gibco, Waltham, MA, USA) supplemented with 10% FBS (Gibco, Waltham, MA, USA), 10 mg/mL penicillin/streptomycin (Gibco, Waltham, MA, USA). As the cells grew in suspension, a ratio of 10^5^ monocytes/mL was maintained in the culture medium for 3 days. The cells were kept in culture flasks and incubated at 37 °C in a 5% CO_2_ atmosphere.

### Cell viability assay

Cell viability was determined using the 3-[4,5-dimethylthiazole-2-yl]-2,5-diphenyltetrazolium bromide (MTT) (Sigma Aldrich, St Louis, MO, USA) assay [22]. First, the endothelial cells were plated in a 96-well plate at a density of 10^4^ cells/well. After 24 h, the cells were treated for 24 h, as described previously. The cells were then incubated with MTT solution (0.5 mg/mL) and culture medium for 4 h at 37 °C. The medium was replaced with dimethyl sulfoxide (DMSO), and the absorbance was measured at 570 nm (Bio-Rad, Hercules, CA, USA). The results are expressed as % control (untreated cells) of three independent biological replicates, each with five technical replicates.

### Cell proliferation assay

Cell proliferation was evaluated using violet crystal coloration. The cells were initially plated in a 96-well plate at a density of 10^4^ cells/well. After 24 h, the cells were treated for 24 h as described previously. The cells were fixed with methanol 100% for 10 min at 25 °C and incubated with violet crystal (0.25 mg/mL) for 20 min at 25 °C. Then, the cells were washed twice with phosphate buffered saline (PBS), eluted with acetic acid 33% and the absorbance was measured at 570 nm (Bio-Rad, Hercules, CA, USA). Three independent biological replicates, each with five technical replicates were conducted for this experiment.

### Gene expression

Total RNA was isolated from the cells lysed with TRIzol (Invitrogen, Waltham, MA, USA) according to the manufacturer’s instructions. RNA integrity was analyzed by 1% agarose gel electrophoresis, and purity and concentration by the absorbance ratio measured at 260 nm and 280 nm on a NanoDrop 2000 spectrophotometer (Thermo Fisher Scientific, Waltham, MA, USA). The mRNA was converted into complementary DNA (cDNA) using a High Capacity RNA-to-cDNA kit (Applied Biosystems, Foster City, CA, USA) according to the manufacturer’s instructions. The cDNA was amplified with specific primer oligonucleotides (Table 1) and SYBR™ Green PCR Master Mix (Applied Biosystems, Foster City, CA, USA) on the StepOnePlus™ Real-Time PCR System (Applied Biosystems, Foster City, CA, USA). Relative gene expression was analyzed by the 2^−ΔΔCT^ method [23] using hypoxanthine phosphoribosyltransferase (*HPRT*) as endogenous control. Results are based on three independent biological replicates, each with two technical replicates.

**Table 1:** Oligonucleotides used in RT-qPCR analysis

Gene	Forward primer (5’-3’)	Reverse Primer (5’-3’)
*HPRT*	GAACGTCTTGCTCGAGATGTGA	TCCAGCAGGTCAGCAAAGAAT
*IL-6*	GACAGCCACTCACCTCTTCA	AGTGCCTCTTTGCTGCTTTC
*IL-8*	CTGCGCCAACACAGAAATTATTGTA	TTCACTGGCATCTTCACTGATTCTT
*IL-1β*	ATGATGGCTTATTACAGTGGCAA	GTCGGAGATTCGTAGCTGGA

Legend: HPRT, hypoxanthine phosphoribosyltransferase; IL, interleukins

### Concentration of systemic and vascular inflammatory biomarkers

Protein levels of IL-6, IL-8, IL-1β, TNF-α, VEGF-A and SAA were measured in the US pool and cell supernatant of treated cells using a ProcartaPlex™ Multiplex Immunoassay (Thermo Fisher Scientific, Waltham, MA, USA #Cat. Number PPX-06-MXYMKPU). TGF-β levels were measured using a ProcartaPlex™ Human Basic Kit (Thermo Fisher Scientific, Waltham, MA, USA #Cat. Number EPX010-10420-901). ICAM-1 protein levels were measured using a Human ICAM-1 Platinum ELISA (Thermo Fisher Scientific, Waltham, MA, USA #Cat. Number BMS201). MCP-1 protein levels were evaluated in cell supernatant samples using a ProQuantum Human MCP-1 Immunoassay Kit (Thermo Fisher Scientific, Waltham, MA, USA #Cat. Number A35598). Three independent biological replicates were conducted for this experiment.

### Monocyte transmigration assay

Cell migration assays were performed using modified Boyden chambers with a 6.5 mm diameter, 10 μm thickness, and porous (5.0 μm) polystyrene membrane separating the two chambers (GE Healthcare Bio-Sciences, Westborough, MA, USA). The endothelial cells were initially treated as described previously, and the cell supernatant was added to the lower compartment of the chamber to evaluate the chemoattractant effect of this supernatant on the monocytes. Next, 10^6^ U-937 cells/mL were added to the top of the chamber over the membrane. The cells were incubated at 37 °C and 5% CO_2_ for 90 min. The chemoattractant ability of the supernatant (migration) was measured by counting the number of cells that were in the lower chamber compartment with a Neubauer chamber. The results are based on three independent biological replicates, each with two technical replicates.

### Scanning Electronic Microscopy

For scanning electronic microscopy analysis, 10^5^ cells were fixed on microscope slides for 1 h in Karnovsky solution (2% glutaraldehyde, 4% paraformaldehyde, 1 mM CaCl_2_ in 0.1 M sodium cacodylate buffer), and post-fixed with 1% osmium tetroxide for 1 h. The cells were dehydrated, then subjected to the critical point in the CPD 010 (Critical Point Dryer) and plated with gold in a SCD 030 apparatus (Balzers Union, Liechtenstein). Images were taken under a scanning electron microscope (Tescan-Vega3-LMU-Scanning electron microscope, Kohoutovice, Czech Republic).

### Statistical analysis

The Shapiro-Wilk normality test was used. For normally distributed data, comparisons between two groups were performed using Student’s *t*-test, whereas one-way ANOVA followed by Dunnett’s post-hoc test or two-way ANOVA followed by Tukey’s multiple-comparisons test was used for comparisons among multiple groups, as appropriate. A *P* < 0.05 value between groups was considered significant. Data are presented in box plots, with individual dots representing the distribution of the individual values. Data were analyzed using the GraphPad Prism 9.3.0 statistical package (GraphPad Software Inc., La Jolla, CA, USA, 2021).

## Results

### Patient characterization

The clinical and biochemical patient data as well as uremic serum pool characterization are described in Table 2.

**Table 2:** Clinical and laboratory characteristics of the study population

Analyzed parameter	Value
Patients (n)	14
Age, years ^*^	55.5 (50.50-65.25)
Race, Caucasian %	64.2
Gender, % male	53
CKD etiology (%)	
Hypertension	13.3
Diabetes	46.7
Glomerulonephritis	26.7
Biochemical data **	
Hemoglobin (g/dL)	12.0 ± 1.3
Hematocrit (%)	36.5 ± 4.1
Albumin (g/dL)	4.1 ± 0.2
WBC (mm^3^)	7.625 ± 2.225
hs-CRP (mg/dL)	0.25 (0.14–1.1)

Legend: WBC, white blood cells; hs-CRP, high-sensitive C-reactive protein. ^*^Values are expressed as number (percentage) or median (25th to 75th percentile). ^**^Values are expressed as the mean ± standard deviation

Next, we measured the biomarker circulation levels involved in endothelial dysfunction and cardiovascular damage present in the uremic serum pool (US) (Table 3).

**Table 3:** Characterization of inflammatory biomarkers in US pool used in cellular treatments.

Molecule	Pool concentration (pg/mL)
IL-6	67.438
IL-8	151.878
IL-1β	7.243
TNF-α	4.697
VEGF-α	3,657.340
SAA	1,946,427.599
TGF-β	10,066.97
ICAM-1	239.958
MCP-1	21.2658

Abbreviations: IL, interleukins; TNF, tumor necrosis factor; VEGF, vascular endothelial growth factor; SAA, serum amyloid A; TGF, transforming growth factor; ICAM; intercellular adhesion molecule; MCP-1, monocyte chemoattractant protein 1

### CRV dose-response curve

**Fig. 1:**
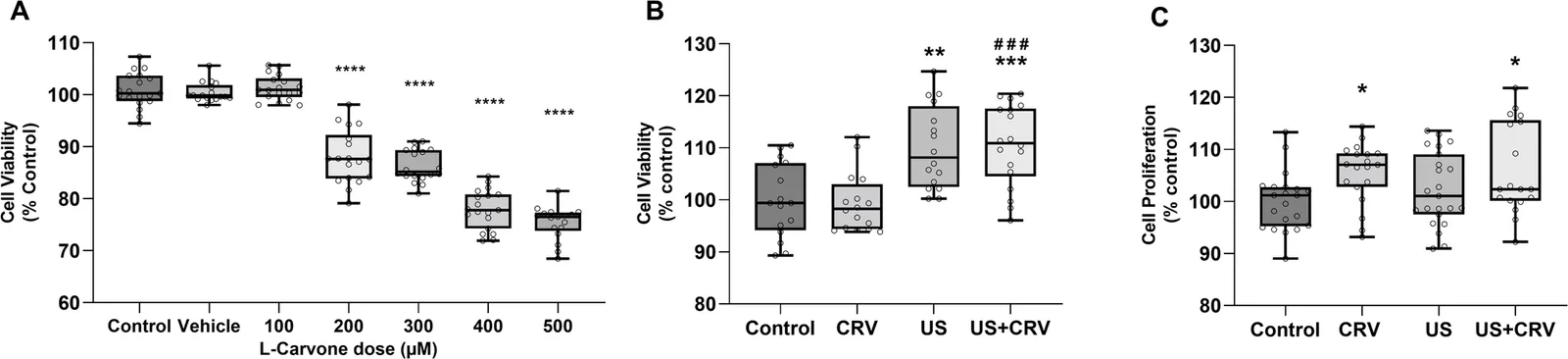
Effects of L-carvone treatment in endothelial cells. **(A)** L-carvone dose–response curve at different concentrations. Data represents three independent biological replicates with five technical replicates each (*N* = 15 technical replicates per group). One-Way Anova following Dunnett’s post-hoc test. **** *P* < 0.0001: Control vs. 200 µM, 300 µM, 400 µM, 500 µM. **(B)** Effects of CRV, US and US + CRV treatments on endothelial cell viability. Data represents three independent biological replicates with five technical replicates each (*N* = 15 technical replicates per group). Two-Way Anova following Tukey’s post-hoc test. **P < 0.01: Control vs. US; ***P < 0.001: Control vs. US + CRV; ^###^*P* < 0.001: CRV vs. US + CRV. **(C)** Effects of CRV, US and US + CRV treatments in endothelial cell proliferation. Data represents three independent biological replicates with seven technical replicates each (*N* = 21 technical replicates per group). Two-Way Anova following Tukey’s post-hoc test. *P < 0.05: Control vs. CRV, US + CRV. Data are presented as box plots (median and interquartile range; whiskers indicate minimum and maximum values), with individual dots representing individual observations. Abbreviations: control, untreated cells (cells with DMEM only); vehicle, cells treated with 0.01% DMSO; CRV, L-carvone; US, uremic serum pool

A dose–response curve of CRV was performed to evaluate endothelial cell viability. We observed that the vehicle (0.01% DMSO) did not affect cell viability (Fig. 1A). CRV significantly decreased cell viability (*P* < 0.0001) at concentrations of 200, 300, 400, and 500 µM (Fig. 1A). CRV at a concentration of 100 µM notably did not impact cell viability, and do it was chosen for the subsequent experiments.

### Cell viability

Cell viability significantly increased in cells treated with US (*P* < 0.01) when compared with control (untreated) cells. Cell viability increased in cells treated with US + CRV compared with control (*P* < 0,001) and in CRV treated cells compared with US + CRV treated cells (*P* < 0.001). CRV alone did not cause significant changes in cell viability (Fig. 1B).

### CRV promotes cell proliferation

We assessed cell proliferation using the violet crystal assay. Cells treated with CRV or US + CRV presented a significative increase in cell proliferation when compared to control cells (*P* < 0.05) (Fig. 1C).

### Inflammatory biomarkers decreased using CRV in uremic conditions

**Fig. 2:**
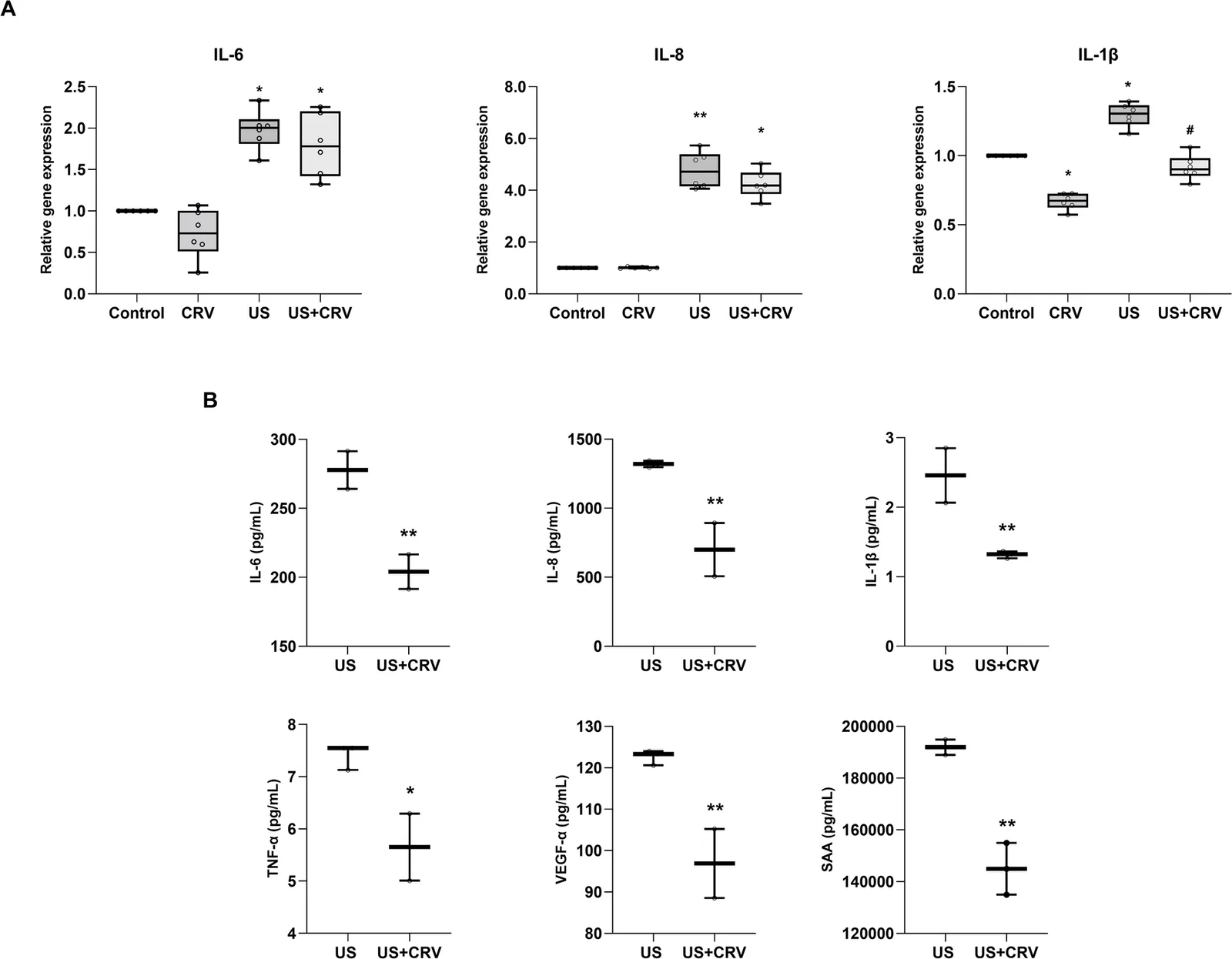
Effects of CRV treatment on inflammatory biomarkers. **(A)** Effects of CRV, US and US + CRV on interleukin gene expression of endothelial cells. Data represents three independent biological replicates with two technical replicates each (*N* = 6 technical replicates per group). Two-Way Anova following Tukey’s post-hoc test. IL-6: *P < 0.05: Control vs. US, US + CRV. IL-8: **P < 0.01: Control vs. US; *P < 0.05: Control vs. US + CRV. IL-1β: *P < 0.05: Control vs. CRV, US; ^#^*P* < 0,05: US vs. US + CRV. **(B)** Effect of CRV on inflammation biomarker (IL-6, IL-8, IL-1β, TNF-α, VEGF-α and SAA) production by endothelial cells in uremic conditions. Data represents three biological replicates with one technical replicate each (*N* = 3 technical replicates per group). Unpaired t-test were used for all targets. IL-6, IL-8, IL-1β, VEGF-α and SAA: **P < 0.01: US vs. US + CRV. TNF-α: *P < 0.05: US vs. US + CRV. Data are presented as box plots (median and interquartile range; whiskers indicate minimum and maximum values), with individual dots representing individual observations. Abbreviations: Control, untreated cells (cells with DMEM only); US, uremic serum; CRV, L-carvone; IL, interleukin; TNF, tumor necrosis factor; VEGF, vascular endothelial growth factor; SAA, serum amyloid A

We evaluated gene expression of interleukins 6, 8 and 1β (IL-6, IL-8 and IL-1β) involved in endothelial inflammation to investigate the potential role of CRV as an anti-inflammatory agent in the endothelial environment (Fig. 2A). For this purpose, endothelial cells were exposed to CRV, US, and US + CRV for 24 h. IL-6 gene expression significantly increased in US and US + CRV treatments compared to control (untreated cells) (*P* < 0.05). US increased IL-8 gene expression (*P* < 0.01), as well as US + CRV(*P* < 0.05). In addition, US increased IL-1β gene expression (*P* < 0.05) compared to control. On the other hand, CRV significantly decreased IL-1β gene expression (*P* < 0.05) compared to control, while US + CRV decreased IL-1β expression compared to US only (*P* < 0.05).

We assessed protein expression by measuring molecules related to inflammation in supernatant of cells treated with US and US + CRV (Fig. 2B). CRV significantly decreased (P < 0.01) endothelial cell production of IL-6, IL-8, and IL-1β, tumor necrosis factor α (TNF-α) production (P < 0.05), as well as decreased vascular endothelial growth factor α (VEGF-α) and serum amyloid A (SAA) (Fig. 2B). Intercellular Adhesion Molecule 1 (ICAM-1) levels were 12.0537 pg/mL and 6.25 pg/mL in US and US + CRV treated cells, respectively

### CRV reduced monocyte recruitment in uremia

**Fig. 3:**
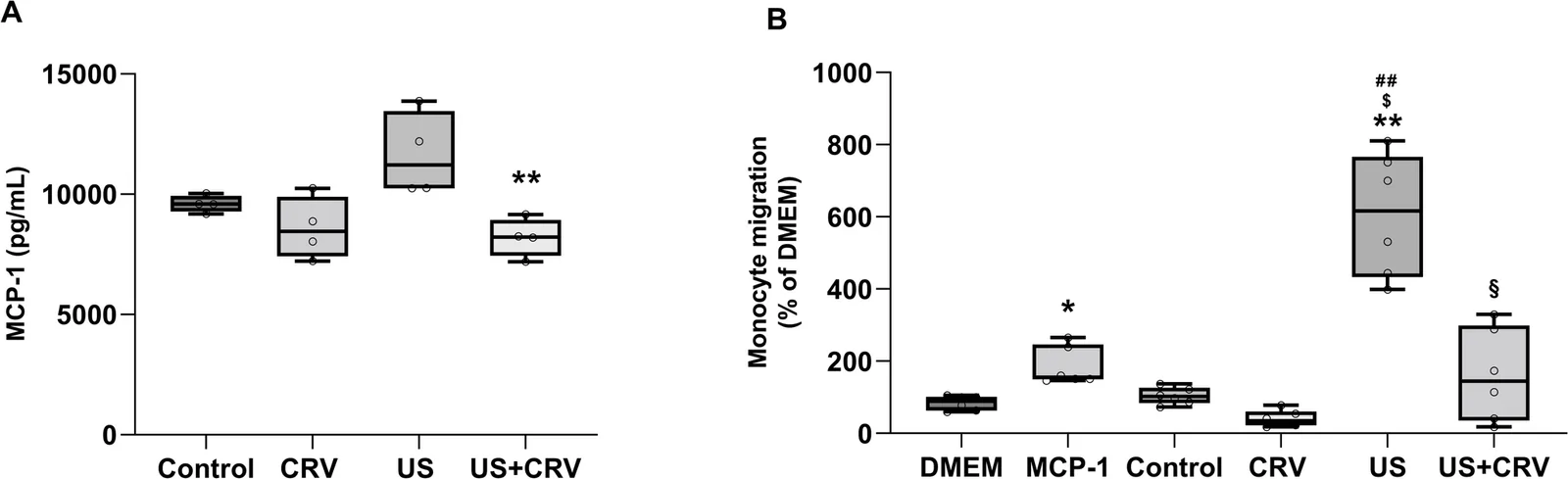
Effects of CRV on MCP-1 production by endothelial cells and monocyte recruitment at uremic conditions. **(A)** MCP-1 measured in supernatant of endothelial cells. Data represents two independent biological replicates with two technical replicates each (*N* = 4 technical replicates per group). Two-Way Anova following Tukey’s post-hoc test. **P < 0.01: US vs. US + CRV. **(B)** Effect of endothelial cell supernatants on monocyte migration. Data represents three independent biological replicates with two technical replicates each (*N* = 6 technical replicates per group). One-Way Anova following Dunnett’s post-hoc test. *P < 0.05: DMEM vs. MCP-1; **P < 0.01: DMEM vs. US; ^$^*P* < 0.05: MCP-1 vs. US; ^##^*P* < 0.01: Control vs. US; ^§^*P* < 0.05: US vs. US + CRV. Data are presented as box plots (median and interquartile range; whiskers indicate minimum and maximum values), with individual dots representing individual observations. Abbreviations: DMEM, Dulbecco’s Modified Eagle Medium; MCP-1, monocyte chemoattractant protein 1; Control, untreated cells (cells with DMEM only); CRV, L-carvone; US, uremic serum

We assessed the MCP-1 measurement of treated endothelial cell supernatant to evaluate monocyte recruitment by endothelial cells. The results showed that CRV reduced MCP-1 production in US + CRV (*P* = 0.0063) compared to US only (Fig. 3A), but CRV did not affect MCP-1 production. Moreover, US increased MCP-1 production, but not significantly. Figure 3B illustrates the effects of endothelial treated-cell supernatant in monocyte migration. DMEM was used as negative control to evaluate spontaneous monocyte migration. MCP-1 was used as positive control (50 ng/mL).

 MCP-1 significantly increased monocyte migration compared to DMEM alone (P < 0.05), as well as US compared to DMEM (P < 0.01). In turn, US significantly increased cell migration compared to MCP-1 (P < 0.05). A significant increase in monocyte migration was noted in US supernatant compared to control (untreated cells) (P < 0.01), while CRV reduced monocyte migration in uremic conditions (US compared to US + CRV) (P < 0.05). The control (untreated cells) and CRV did not affect monocyte migration

### CRV reduced endothelial damage caused by uremia

**Fig. 4:**
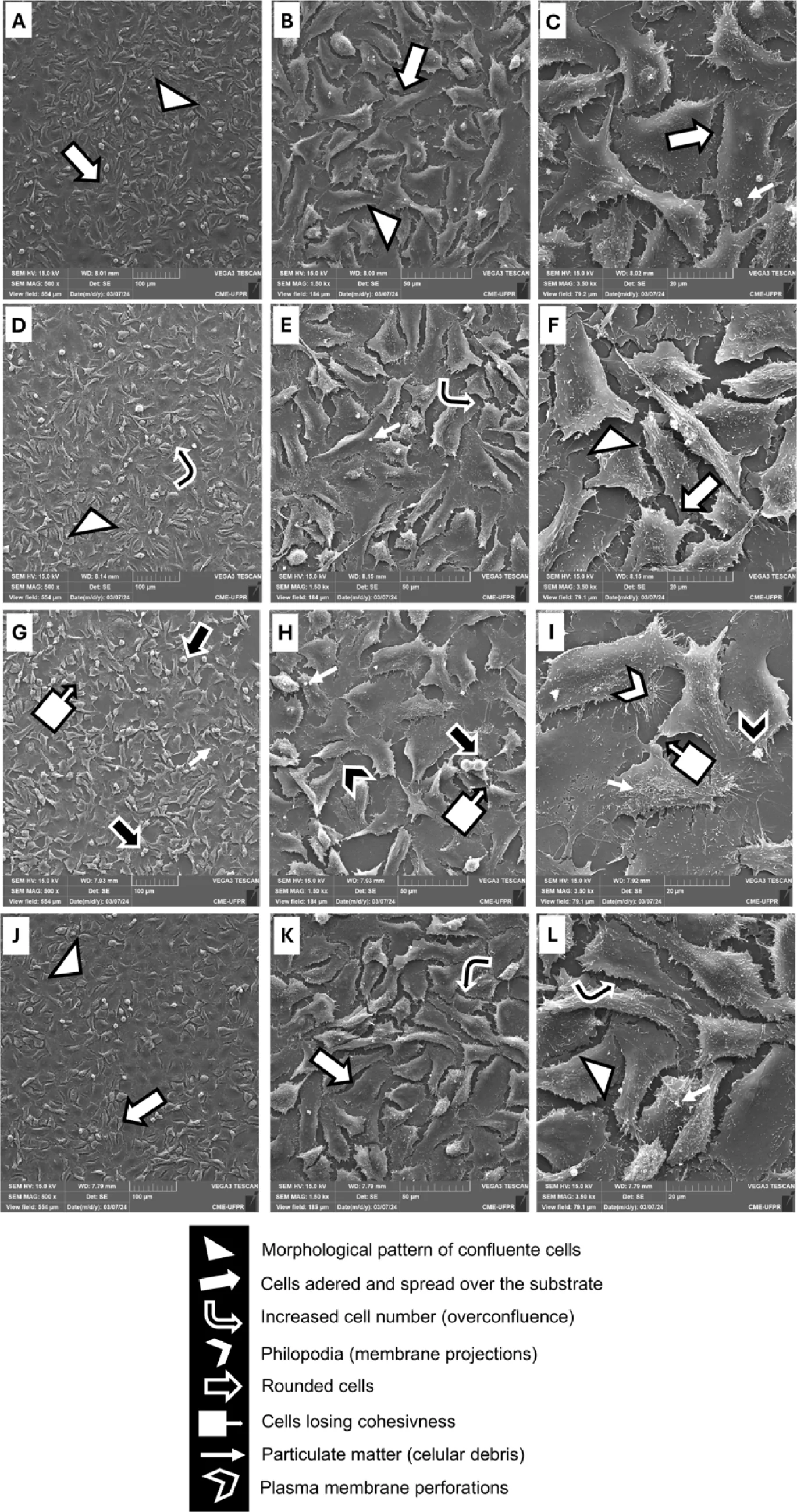
Morphological and ultrastructural analyses of endothelial cells by scanning electronic microscopy. (A-C) Control cells (untreated cells); (D-F) Cells treated with CRV; (G-I) US; (J-L) US + CRV. Magnifications of the images: A, D, G, J: 500X; B, E, H, K: 1500X; C, F, I, L: 3500X. Abbreviations: Control, untreated cells (cells with DMEM only); CRV, L-carvone; US, uremic serum

We conducted scanning electronic microscopy analysis to observe morphological and ultrastructural changes in endothelial cells exposed to US and CRV. Figures 4 A-C shows control cells (untreated cells) adhered to and spread over the substrate in a confluence pattern of monolayer, typical of intact endothelium. Cells exposed to CRV have a similar pattern to control, however with an increased cell number in an over confluence pattern (Figs. 4D-E). Figures 4G-I exhibit endothelial cells treated with US in which morphological changes caused by uremia can be observed, including an increased number of filopodia, loss of intercellular adhesions, rounded cells, perforations in plasma membrane and amounts of cellular debris. However, cells treated with US + CRV (Figs. 4 J-L) display a control-like and CRV-like pattern, with cells adhered to and spread over the substrate and an increased cell number (over confluence and increased number of cellular debris).

## Discussion

CKD is a systemic and widely distributed disease, strongly linked to diabetes and hypertension. As CKD progresses, the vascular endothelium is profoundly affected by an accumulation of uremic toxins, leading to CVD development due to an endothelial exacerbated inflammatory phenotype. In this study, we investigated the in vitro effects of CRV as a potential anti-inflammatory molecule in the context of a uremic environment using human endothelial cells. We observed for the first time that CRV was able to: (I) promote cell proliferation; (II) reduce inflammatory biomarker production; (III) reduce monocyte recruitment and (IV) reduce structural damage caused by uremia. Taken together, these findings suggest the role of CRV in attenuating endothelial inflammatory response caused by uremia in vitro.

This study comprised 14 end-stage CKD patients in hemodialysis. The prevalence of diabetes mellitus, hypertension and glomerulonephritis is associated with aggravation of CKD and development of CVD. The inflammatory biomarker levels in serum such as IL-6, IL-8 and MCP-1 are consistent with previous findings in other studies [6, 24]. These molecules play a central role in driving systemic inflammation in these patients [25]. Other biomarkers like TGF-β are related to renal fibrosis and endothelial damage, including epithelial-mesenchymal transition and arterial calcification [26, 27].

The concentration of CRV (100 µM) used in this study was selected based on cell viability assays and previous in vitro reports describing its biological activity [16, 28]. Previous human studies have demonstrated systemic exposure to CRV following oral administration of CRV-containing preparations, particularly peppermint and caraway oil formulations, as well as absorption following topical exposure [29, 30]. However, these studies do not establish the clinical efficacy or safety of isolated CRV in patients with CKD. Although this concentration may exceed expected circulating levels, available pharmacokinetic studies indicate that CRV is rapidly metabolized and cleared, resulting in low systemic exposure, while quantitative data on achievable plasma or tissue concentrations in humans remain limited [31]. Accordingly, the present findings should be interpreted as mechanistic observations rather than as directly reflective of in vivo exposure.

First, we evaluated the effects of CRV (100 µM) on endothelial cell viability and proliferation. Under these conditions, CRV alone or combined with US did not affect cell viability, whereas US alone increased cell viability. This finding may be attributed to the presence of different metabolites in the serum (i.e. VEGF), which is enhanced in renal patients [32]. Other studies suggest that US does not significantly alter the cell viability profile [8]. CRV and US + CRV also significantly increased cell proliferation. However, increased proliferation alone does not establish endothelial regeneration or restoration of barrier function, which would require specific functional assessments. In the context of uremia, promoting regeneration of the endothelial barrier of the endothelium damaged by an accumulation of uremic toxins can be understood as beneficial [33]. Studies have shown that CRV has antiproliferative properties in cancer models [34]. However, this effect was not observed in control cells, indicating that its action might depend on cellular context.

The exacerbated production of inflammatory molecules by endothelial cells caused by exposure to US is demonstrated by the increased gene expression of IL-6, IL-8, and IL-1β, which agrees with previous studies [6, 35]. CRV effects on the reduction of IL-8 and IL-6 gene expression are not significant; nevertheless, CRV significantly reduces the expression of IL-1β in cells treated with US + CRV, consistent with previous reports [36, 37]. IL-1β plays a key role in enhancing inflammation [38, 39] through activation of the nuclear factor kappa-B (NF-kB) pathway [40], which leads to vascular damage and increased arterial calcification [41]. Notably, CRV reduces the protein expression of inflammatory markers in comparing US and US + CRV.

TNF-α, SAA and VEGF levels were also reduced in response to the combination of CRV and US compared with US alone. TNF-α is an inflammatory mediator that activates endothelial cells and recruits leukocytes [40]. SAA is an acute-phase protein secreted by the liver that is involved in inflammatory responses triggered by ILs [42, 43]. VEGF increases vascular permeability and reduces endothelial stability, accentuating endothelial dysfunction [44]. Reducing the levels of these markers may indicate an attenuation of the endothelial inflammatory response under uremic conditions; however, whether this translates into reduced systemic inflammation in CKD patients remains to be determined. Studies showed that CRV significantly reduces the production of interleukins and chemokines in vivo [45]. Although these effects have been associated with inhibition of the NF-κB signaling pathway in other experimental models [28, 46], we did not directly assess NF-κB activation or antioxidative pathways. Therefore, the involvement of these mechanisms in the observed effects of CRV remains speculative and warrants further investigation [42, 43]. These proteins are also involved in leukocyte recruitment, producing adhesion molecules like ICAM-1, and forming atherosclerotic plaques [47]. The reduction of these proteins has been observed through treatment with other monoterpenes in vivo, suggesting that CRV may attenuate inflammatory responses associated with vascular injury [48]. Monoterpenes have shown potential in reducing the expression of adhesion molecules and altering the progression of atherosclerosis [18].

Monocyte recruitment and MCP-1 production are reduced by CRV treatments, in agreement with previous findings with other natural molecules [49]. In a synergistic mechanism, the decrease of inflammatory molecules and leukocyte recruitment is associated with attenuating systemic inflammation [50]. Studies have shown that the reduction of these markers is linked to a better prognosis regarding CVD [25].

Uremia-induced morphological alterations in endothelial cells play a central role in endothelial disfunction by disrupting intercellular junctions and increasing cell permeability [8]. In our experimental model, CRV was associated with attenuation of these morphological alterations and increased cell proliferation. Although increased proliferation may be compatible with processes involved in endothelial repair, the present study did not directly assess endothelial barrier integrity, vascular function, or revascularization. Therefore, these findings should not be interpreted as evidence of endothelial regeneration or vascular protection.

We recognize that our study has limitations and that further work is necessary. The mechanisms involved in NF-kB modulation by CRV need to be explored, as well as its antioxidative role. We used the EA.hy926 cells as an endothelial study model. Although these cells are well established for in vitro studies, they may not fully recapitulate the phenotype and functional responses of primary human endothelial cells, warranting validation in primary cells. It would also be interesting to evaluate the effects of CRV in other cell types, such as smooth vascular cells and in renal tissue. The use of pooled serum from 14 patients represents another limitation, as it does not capture inter-individual variability among CKD patients and may limit generalizability. Studies using serum from a larger number of individual patients will be important to determine whether the observed effects are consistent across different patient profiles. In addition, studies in vivo will be necessary to provide valuable insights into the effects of CRV in complex biological systems.

## Conclusions

In summary, we demonstrated for the first time that the dysfunctional and inflammatory profile of endothelial cells caused by uremia seems to be attenuated by CRV treatment. This study presents a potential beneficial role of CRV in uremia by reducing the expression of inflammation biomarkers, reducing leukocyte recruitment, and reverting endothelial damage caused by uremia. Taken together, these data highlight the potential of CRV in mitigating vascular damage and preventing worsening of endothelial dysfunction in CKD, however further studies are required to determine the translational relevance of these findings. Our study opens perspectives to evaluate the involvement of CRV in uremic inflammation process to better understand the mechanisms involved in vascular injury in CKD patients and targets to reduce endothelial damage.

## Data Availability

No datasets were generated or analysed during the current study.

## Abbreviations

CKD: chronic kidney disease
CRV: L-carvone; CVD: cardiovascular diseases
ICAM-1: intercellular adhesion molecule-1
IL: interleukin
MCP-1: monocyte chemoattractant protein 1
SAA: serum amyloid A
TNF: tumor necrosis factor
US: uremic serum
VEGF: vascular endothelial growth factor
